# The regulation of sulfolipids under sulfur starvation

**DOI:** 10.1007/s11103-023-01364-2

**Published:** 2023-06-22

**Authors:** Fayezeh Aarabi, Mohamed A. Salem, Stephanie Arrivault, Mustafa Bulut, Mark Aurel Schöttler, Patrick Giavalisco, Alisdair R. Fernie, Rainer Hoefgen

**Affiliations:** 1 Max-Planck-Institute of Molecular Plant Physiology, Am Mühlenberg 1, Golm, 14476 Potsdam, Germany; 2 Department of Pharmacognosy and Natural Products, Faculty of Pharmacy, Menoufia University, Gamal Abd El Nasr St, Shibin Elkom, 32511 Menoufia Egypt; 3 Max Planck Institute for Biology of Ageing, Joseph Stelzmann Str. 9b, 50931 Cologne, Germany

## Abstract

Despite the fact that sulfolipids comprise a mere 4–7% of total leaf lipids, as demonstrated here, they are functionally crucial under certain environmental conditions, such as phosphate and sulfate deficiencies.

**Supplementary Information:**

The online version contains supplementary material available at 10.1007/s11103-023-01364-2.

Dear Editor,

In previous studies, it was identified that the Sulfur deficiency-induced proteins SDI1 and SDI2 play a fundamental role in sulfur homeostasis under sulfate-deprived conditions (−S) by downregulating two main sulfur pools in *Arabidopsis*, namely the glucosinolates (GSL; S-rich secondary metabolites) (Aarabi et al. 2016) and S-rich 2S seed storage proteins in *Arabidopsis* seeds (Aarabi et al. 2021) (Fig. 1a). Furthermore, the SDI inhibitory effect on GSL and seed proteins was deciphered to be carried out by inhibition of MYB28 and MYC2 TFs. The results of our current study indicate that SDI1 not only down-regulates GSL and 2S-seed storage protein accumulation under –S but also specifically down-regulates sulfolipid, sulfoquinovosyldiacylglycerol (SQDG) levels (Fig. 1a and d), with levels of other chloroplastic membrane lipids being unaltered dataset S1).

**Fig. 1:**
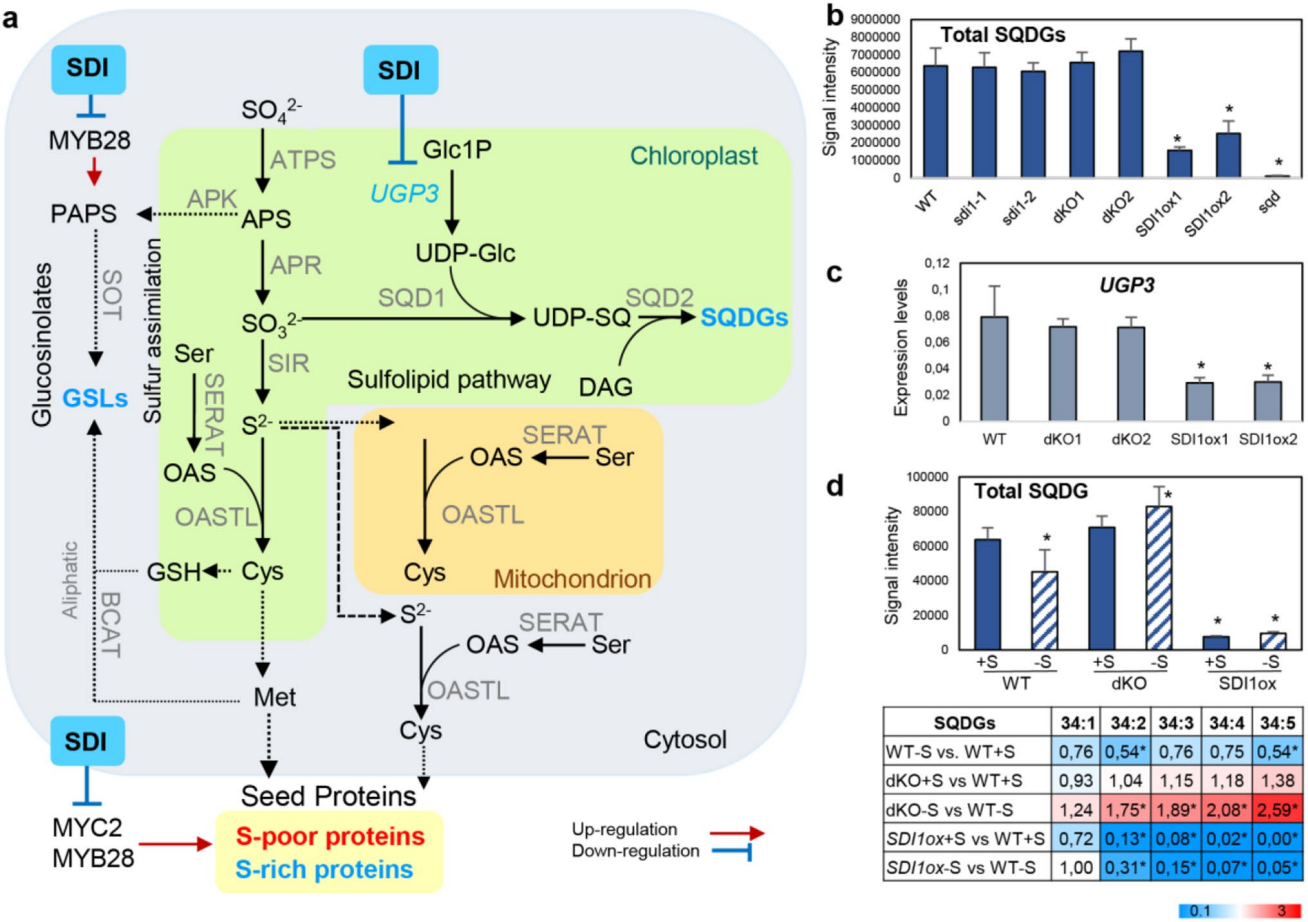
**a** Sulfur assimilation pathway and its derivative metabolites. The main branching pathways, glucosinolates (GSL), sulfolipids (SQDG), and sulfur-containing proteins, are depicted. There are only three enzymes responsible for SQDG biosynthesis in the stroma of chloroplasts, UDP—glucose pyrophosphorylase (UGP3), UDP-sulfoquinovose (UDP-SQ) synthase (SQD1), and SQDG synthase (SQD2). In addition, the role of SDI on the inhibition of the TF or genes of the corresponding branching pathways is portrayed. **b** Sulfolipid contents of the soil-grown six-week-old transgenic plants. (mean + SD of five biological replicates), *Dunnett test adjusted p-value < 0.05 versus wildtype, (five biological replicates). Full lipidomics is presented in supplementary dataset S1. **c** Expression levels of *UGP3* of the plants as described in b. Transcript levels relative to the internal control gene *Ubiquitin* 10 (*UBQ10*) were quantified by qRT-PCR (mean ± SD of 3 biological and 2 technical replicates, 2^−(ct*UGP3*−ct*UBQ*)^) and is represented for WT, dKO, and SDI1ox lines. Additionally, the fold change expression level of *UGP3* relative to WT is represented in Fig. S2b by calculating 2^−(ct*UGP3*−ct*UBQ*)−(ct*WT*−ct*UBQ)*^*.* *Two tailed T-test P < 0.05 vs wildtype. **d** SQDG contents of the transgenic lines grown under + S and –S conditions. *Arabidopsis* seedlings were grown in sulfur-sufficient medium (+ S/400 μM) in liquid culture for seven days and then subjected to zero sulfur (−S/0 μM) medium for 2 days. Heatmap showing the differential behavior of SQDG molecular species in *dKOs* and *SDI1ox* lines. Each value represents the ratio of each related metabolite versus the corresponding WT. *Dunnett test adjusted p-value < 0.05 versus wildtype at each condition, (four biological replicates). Full lipidomics is presented in supplementary dataset S2

Indeed, lipid measurements (see Supplementary materials for methods) revealed that total SQDG contents in *SDI1*ox lines were significantly reduced to 24–39% of WT levels when grown on soil (Fig. 1b, dataset S1), with some individual SQDG molecular species being even more compromised in the transgenics (Fig. S1, dataset S1). Comparable changes in lipid composition in *SDI1ox* lines were also observed in the *sqd1* mutant whose SQDG levels were also drastically reduced (Fig. S1, dataset S1).

Transcript analyses (see Supplementary material for methods), revealed that SDI1 acts negatively on gene expression of *UGP3* (Fig. 1c), the first enzyme of the SQDG pathway, but does not influence other genes of the pathway (Fig. S2a and b). This result agrees with previous studies where *UGP3* was the only SQDG pathway gene down-regulated in response to short-term sulfur starvation (Okazaki et al. 2009). In addition, *UGP3* mutants were found to be almost devoid of SQDGs under normal and phosphate-depleted growth conditions (Okazaki et al. 2009), pointing to the key role of *UGP3* expression in controlling the sulfolipid metabolism. Nikiforova et al. (2005) showed that, in response to long-term sulfur starvation, the expression of *SQD2* and the levels of SQDG lipids were decreased (Nikiforova et al. 2005). Thus, *UGP3* and *SQD* genes are suggested to be differentially regulated in response to sulfur deficiency in a time-dependent manner (Okazaki et al. 2009). Although the biosynthetic pathway of sulfolipids has been fully deciphered, the regulatory mechanisms by which sulfolipid biosynthesis is controlled have yet to be established. We hypothesize that SDI down-regulates an unknown TF of the pathway that controls *UGP3* expression. However, analysis of *MYB28ox* and *MYB29ox* microarray data revealed that *UGP3* expression is considerably increased, suggesting that *UGP3* may be positively controlled by MYB28 and MYB29 (Sonderby et al. 2007). That said, lipidomics measurements did not reveal any significant changes in the SQDG levels of the *myb* KO and ox lines (Fig. S1, dataset S1), raising the possibility that MYB28, MYB29, and MYB76 may not be positive regulators of *UGP3* and, subsequently, SQDG metabolism. Moreover, some unknown metabolic factors or regulators for *UGP3* may exist that cannot be revealed by transcriptomic analyses; therefore, future investigations are required to decipher the positive regulatory factors of *UGP3*.

However, in lipidomics measurements of *Arabidopsis* seedlings grown in shaking culture, we could confirm our data that *SDI1* overexpression down-regulated SQDG contents with an opposite behavior being observed in *sdi1sdi2* double knockouts (dKOs) (Fig. 1d, dataset S2). Moreover, in shaking cultures, sulfur deficiency amplified the response of SQDGs to *SDI* status. This effect could be observed in dKOs, which accumulated about twofold higher SQDG contents under –S compared to WT (Fig. 1d, dataset S2). Individual SQDG species in *SDI1ox* lines were dramatically reduced under + S and –S conditions, respectively (Fig. 1d, dataset S2). Furthermore, under –S, the *SDI1ox* line displayed a significant increase in total PG levels, mainly attributed by an increase in PG 34:3 (Fig. S3, dataset S2). This observation highlights the role of SDI on SQDG regulation under conditions of low sulfate where SQDGs are more drastically reduced and in turn, PGs are substituted for SQDGs.

Initially, SQDGs were assumed to be essential for photosynthesis (Benning 1998); however, analyses of bacterial and *Arabidopsis* SQDG-mutants revealed only a subtle effect of these lipids on photosynthetic efficiency under normal growth conditions (Yu et al 2002; Okazaki et al 2013). In *Arabidopsis* *sqd2pgp1-1* double knockouts, lacking SQDG and containing low levels of PG, the total level of anionic lipids was considerably reduced, more than in each single mutant. This resulted in reduced chlorophyll contents and photosynthetic capacity, suggesting that anionic lipids are essential for photosynthesis (Yu and Banning 2003; Shimojima 2011).

Given the growth defects of *SDI1ox* lines (Aarabi et al. 2021) and the reduced content of chloroplastic sulfur-containing SQDG lipids in these lines, photosynthetic parameters were quantified in order to determine whether the photosynthetic capacity of the transgenic lines was affected. *SDI1ox* lines together with dKOs and WTs were grown under short-day conditions for six weeks, and chlorophyll a fluorescence parameters were measured using the MAXI-version of the Imaging-PAM. In addition to leaf absorptance, the chlorophyll a fluorescence parameters maximum quantum efficiency of PSII photochemistry (F_V_/F_M_, measured after 30 min of dark adaptation), and linear electron transport capacity (ETRII, derived from a light response curve of linear electron transport), were quantified (Table 1). Moreover, the chlorophyll content per leaf area and the chlorophyll a/b ratio were determined. Data analysis revealed that chlorophyll a/b ratios of all *SDI1ox* lines were significantly lower than WT (Table 1). Furthermore, both *SDI1ox* lines showed a significantly reduced electron transport capacity (Table 1). However, all observed differences were tiny, though significant. In general, despite the growth defects of *SDI1ox* lines, the photosynthetic capacities of the plants were only partially affected. In line with previous studies mentioned above, our data demonstrate that sulfolipids play an essential but minor role in maintaining the photosynthetic capacity of plants (Yu and Benning 2003; Shimojima 2011).

**Table 1:** Photosynthesis parameters measured in transgenic lines and WTs grown for 6 weeks under short-day conditions

Genotype	Chlorophyll a/b	Chlorophyll [mg m^−2^]	Absorptance [%]	F_V_/F_M_	ETRII [µmol m^−2^ s^−1^]
WT	**3,46** ± 0,11	**251,7** ± 17,9	**0,79** ± 0,02	**0,82** ± 0,01	**54,9** ± 3,3
*SDI1ox1*	**3,32** ± 0,08	**221,0** ± 16,8	**0,74** ± 0,04	**0,81** ± 0,00	**46,3** ± 4,2
*SDI1ox2*	**3,25** ± 0,06	**249,1** ± 16,4	**0,76** ± 0,05	**0,81** ± 0,01	**45,7** ± 6,9
*dKO1*	**3,45** ± 0,10	**228,2** ± 18,7	**0,75** ± 0,02	**0,82** ± 0,01	**53,6** ± 4,3
*dKO2*	**3,45** ± 0,09	**245,5** ± 20,4	**0,73** ± 0,03	**0,82** ± 0,01	**52,5** ± 4,2

F_V_/F_M_ shows maximum quantum efficiency of PSII photochemistry in the dark-adapted state. ETRII shows electron transport capacity. Significant differences, relative to WT, are highlighted in Bold. Data were analyzed in SigmaPlot using a one-way ANOVA (Holm-Sidak test)

*n* = 9

So far, the molecular mechanism controlling the reduction of SQDG levels under sulfur stress is not yet determined, and it is not known whether the de novo synthesis of SQDG lipids is reduced or degradation is increased. However, it has been demonstrated in *Chlamydomonas reinhardtii* that SQDG plays a vital role as an internal S-source under -S conditions (Sugimoto, et al. 2007). In this species it has been shown that within 6–12 h after sulfur starvation*,* SQDG almost entirely breaks down, thereupon providing a large proportion of S for the synthesis of proteins (Sugimoto, et al. 2010). Therefore, besides SDI-mediated control of sulfolipids, sulfolipid degradation might occur in plants to replenish exhausted sulfur pools.

In conclusion, the results of our study provide additional knowledge about the regulation of sulfolipids under sulfur starvation. They will likely form a solid basis for future studies of this important class of lipids and a route by which to identify additional sulfolipid control factors. Although SQDG contents are relatively minor compared to the other glycerolipids -MGDGs, and DGDGs—and despite the fact that they comprise a mere 4–7% of total leaf lipids, as demonstrated here, they are functionally crucial under certain environmental conditions, such as phosphate and sulfate deficiencies. SQDGs are suitable substitutes for PGs to maintain the chloroplast functional organization as they have similar biophysical properties (Bolik et al. 2022). Furthermore, given that membrane lipid remodeling is a critical strategy for plants’ adaption to nutrient starvation, SDI appears to play a critical role in this mechanism. Moreover, our study highlights the role of SDI, under –S per se, demonstrating a considerable role in simultaneously down-regulating the three main sulfur pools in plants, GSLs, the seed 2S-rich proteins, and SQDGs. Thus, revealing that this single protein mediates three critical functions in the maintenance of plant homeostasis under sulfate deprivation.

## Supplementary Information

Below is the link to the electronic supplementary material.

Supplementary file1 (DOCX 786 kb)

## Data Availability

Enquiries about data availability should be directed to the authors.
