# Supplementary material for: The regulation of sulfolipids under sulfur starvation
Source: Plant Mol Biol. 2023 Jun 22;112(4-5):195–8. doi: 10.1007/s11103-023-01364-2 (PMC10352420; doi:10.1007/s11103-023-01364-2)
Supplement: Supplementary file 1 — Supplementary file1 (DOCX 786 kb) [file 11103_2023_1364_MOESM1_ESM.docx]

**Supplemental information**


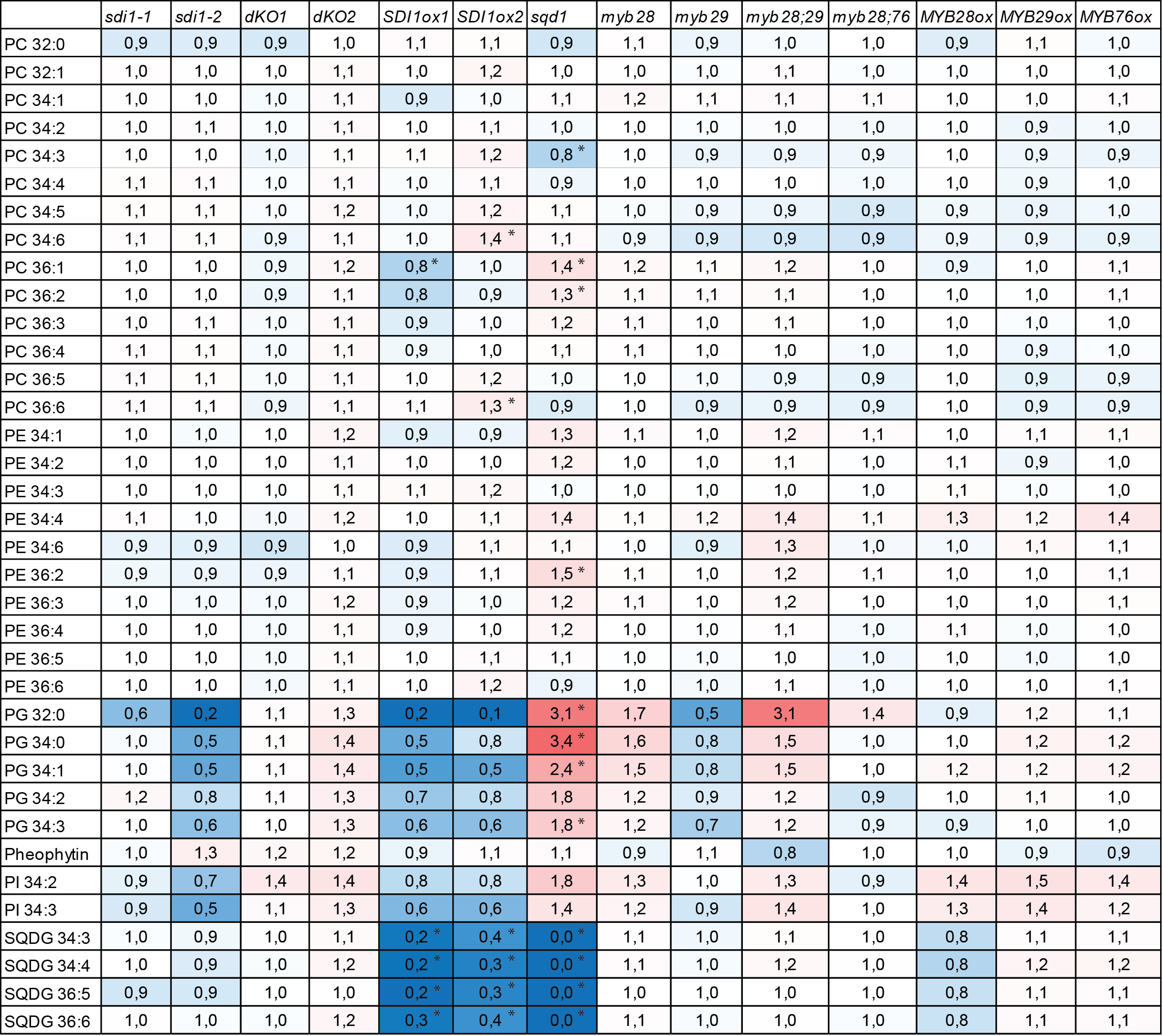


**Fig. S1.** Heatmap showing the differential behavior of phospholipids and sulfolipids of the soil-grown six-week-old transgenic plants. Each value represents the ratio of each related metabolite versus the corresponding WT. Five biological replicates were used. * Dunnett test adjusted p-value <0,05 vs the corresponding wildtypes, (five biological replicates). Full lipidomics is presented in supplementary dataset S1

**
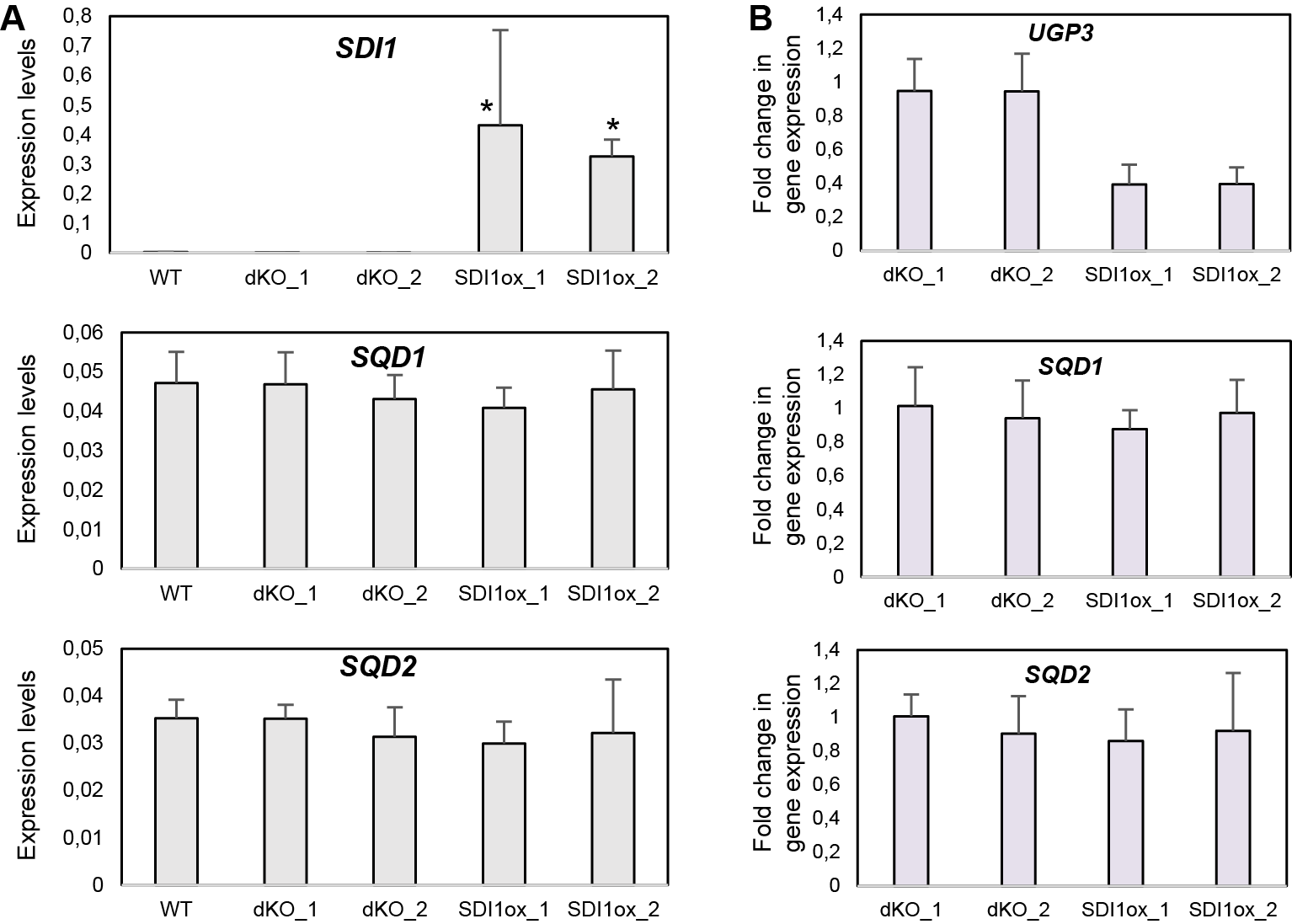
**

**Fig. S2.** Expression level of *SDI1*, *UGP3,* *SQD1*, and *SQD2* of the plants as described in Figure 1B. **a** Transcript levels were quantified by qRT-PCR (mean ± SD of 3 biological and 2 technical replicates, 2^-(ct^ *^gene of inetrest^*^-ct^ *^UBQ^*^)^). * Two tailed T-test P<0,05 vs wildtype. **b** The fold change expression level of *UGP3*, *SQD1*, and *SQD2* relative to WT is represented in Figure S2B by calculating 2^-(ct^*^Gene of interest^*^-ct^*^UBQ^*^)-( ct^*^WT^*^-ct^*^UBQ)^.*


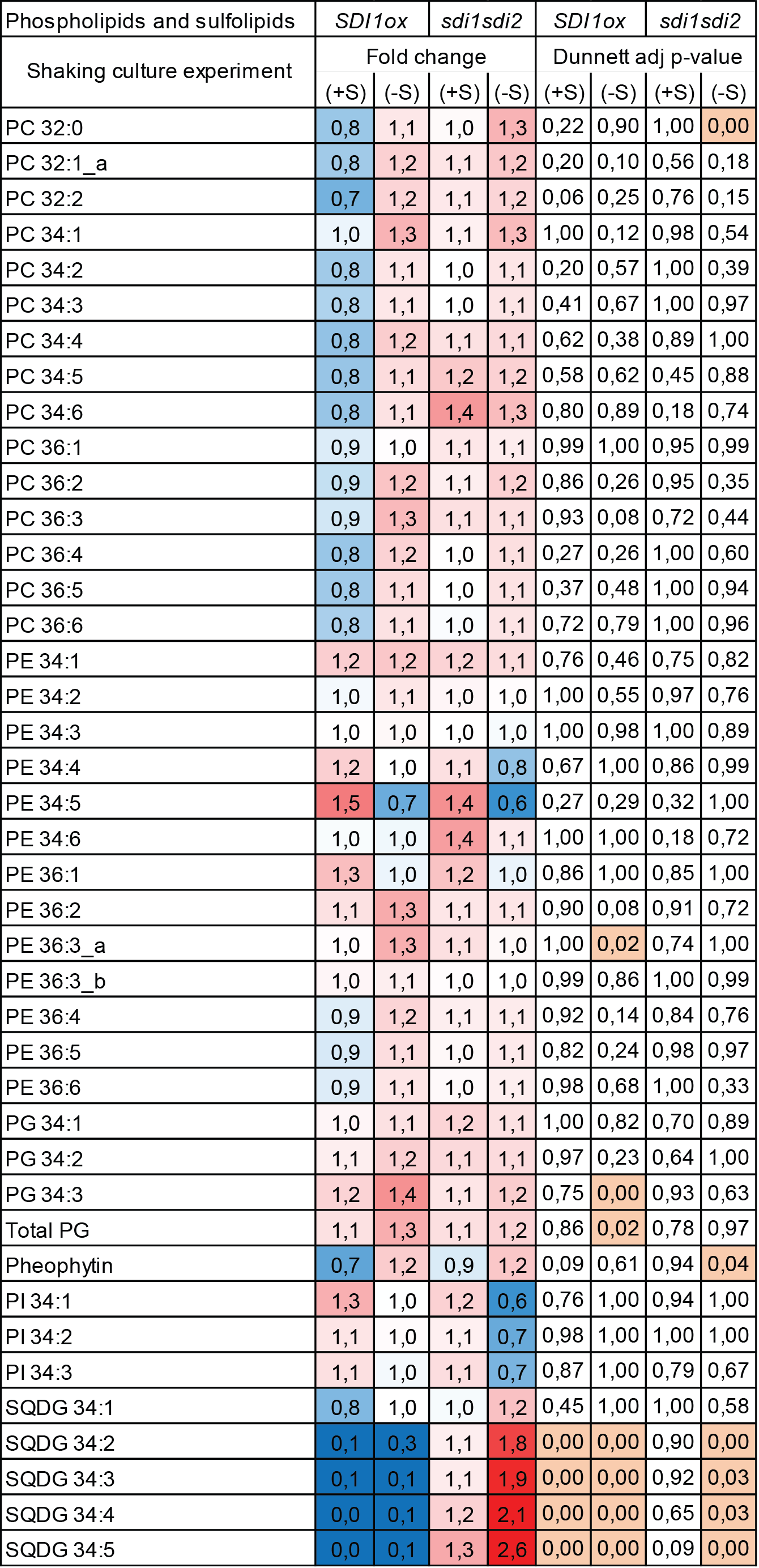


**Fig. S3.** Heatmap showing the differential behavior of phospholipids and sulfolipids in dKOs and *SDI1ox* lines grown under (+S/400 μM) and (-S/0μM) media as descriped in Fig. 1d. Each value represents the ratio of each related metabolite versus the corresponding WT at each condition. * Dunnett test adjusted p-value <0,05 vs wildtype at each condition, (four biological replicates). Full lipidomics is presented in supplementary dataset S2.

**Table S1.** Primers used for the RT-qPCR analysis.

| **Gene name** | **Forward primer (5' to 3')** | **Reverse primer (5' to 3')** |
| --- | --- | --- |
| *UGP3* | TCAAGGGAGGGTCCATTTCCAG | ACGATCAAGCTACCATCAACCTG |
| *SQD1* | TGGTAAAGGTGGTCAGACGAGAG | TCTCAACACATTGAACCGTGTCTC |
| *SQD2* | TGTTGAGCCTTCTCCCTTTGCC | TCAGGAACACCTTCATGTGTCGTC |
| *SDI1* | TCACAAAGTTCCCTGTGGAGACAC | CGGGTTCTTCTCTATCAACTGAGC |
| *UBQ10* | GGCCTTGTATAATCCCTGATGAATAAG | AAAGAGATAACAGGAACGGAAACATAGT |
